# Gait Event Detection From Thigh Segment Kinematics in Children With Crouch Gait

**DOI:** 10.1109/JTEHM.2026.3679585

**Published:** 2026-03-31

**Authors:** Jordan Dembsky, Noah Rubin, Thomas C. Bulea

**Affiliations:** Rehabilitation Medicine DepartmentNational Institutes of Health Clinical Center 24481 Bethesda MD 20892 USA

**Keywords:** Gait events, thigh kinematics, sensors, exoskeletons, crouch gait

## Abstract

Objective: Wearable exoskeletons can improve walking in children with movement disorders by delivering precisely timed and scaled torques based on discrete gait phase. Most single-joint systems segment the gait cycle using underfoot force-sensitive resistors (FSRs), but FSRs are suboptimal for exoskeleton use in pathological gait due to fragile hardware and inconsistent foot loading/unloading. Thigh kinematics may provide more robust gait event detection (GED), yet this approach has not been evaluated in children with crouch gait, a key target population for pediatric exoskeleton therapy. This study aimed to: 1) assess feasibility of GED using thigh kinematics from ground-truth motion capture, 2) evaluate GED using a thigh-mounted inertial measurement unit (IMU), and 3) validate this method with a wearable knee exoskeleton in children with crouch gait.Methods and procedures: A novel thigh segment kinematics algorithm (TSKA) was developed to detect initial contact (IC) and terminal contact (TC) during overground walking. Algorithm performance was assessed using IMU-derived gait events compared to motion capture ground truth, then evaluated with a wearable knee exoskeleton.Results: Mean IMU-based timing errors for IC and TC were 38 and 84 ms, respectively. IC predictions occurred earlier than ground truth, whereas TC predictions varied. During exoskeleton walking, IMU-based GED significantly improved timing accuracy compared to FSR-based detection.Conclusion: The thigh-based kinematic algorithm, tuned for each individual with six or fewer strides, accurately detected gait events in children with crouch gait and outperformed FSR-based GED in an exoskeleton. These findings support further development for real-time exoskeleton control and gait assessment in individuals with pathological gait.

## I. Introduction

Mobile overground exoskeletons have been increasingly used to improve walking in individuals with gait impairments. These devices fall into two main categories: those designed for full mobilization and those offering partial assistance [1]. Full mobilization exoskeletons provide all necessary torque for users with severe motor impairments [2], while partially assistive devices support a portion of required torque for movement in users with less severe or no deficits. Exoskeletons can also apply resistance to facilitate rehabilitative training, offering a promising method for improving strength and coordination [1], [3], [4]. Given the repetitive nature of walking, exoskeletons are particularly well-suited for delivering gait training at high doses while also reducing reliance on clinical staff [5].

Robotic exoskeletons employ a range of control strategies, including trajectory tracking, admittance shaping, and torque controllers [1]. While recent advancements allow task-agnostic control [6], most controllers optimize a specific task or group of tasks. Controllers designed for gait rehabilitation often utilize control laws which either subdivide the gait cycle into discrete phases or model the gait cycle as a continuous variable. The former controller type, a finite state machine (FSM), detects transitions between gait cycle phases, with each phase linked to a specified torque profile, while the latter often specifies torque based on continuous gait phase estimation.

Accurate gait event detection (GED), particularly of initial contact (IC) and terminal contact (TC), is essential for segmenting the gait cycle and timing exoskeleton torque application [7]. While force plates and/or foot-velocity algorithms remain the gold standard for GED in lab environments [8], portable solutions are needed for real-world applications. Common portable approaches include force-sensitive resistors (FSRs) and inertial measurement units (IMUs) [9]. FSRs use threshold-based detection [10], while IMUs can estimate gait events using gyroscopic or accelerometer signals from the thigh, shank, or foot [8], [11]. Each method has its own limitations. FSRs are prone to hysteresis, voltage variability, and mechanical degradation, all of which can affect accuracy and require frequent recalibration or replacement [12], [13]. IMU-based methods suffer from drift, environmental sensitivity, and may need extensive filtering [14]. These challenges are amplified in individuals with atypical gait, such as those with crouch gait, due to deviations in joint kinematics and asymmetrical movement patterns [15]. Given the importance of accurate GED in exoskeleton control, these limitations pose challenges for device performance and therapeutic outcomes.

Rehabilitative exoskeletons have been used for several gait disorders, including cerebral palsy (CP), one of the most common causes of childhood disability [5]. Crouch gait, a common pathology in CP, is characterized by excessive hip and knee flexion during stance and insufficient knee extension during mid-stance and late swing [16]. Left untreated, it can lead to worsening mobility and loss of ambulation [17]. Interventions such as orthopedic surgery, strength training, and botulinum toxin injections are commonly used but offer limited long-term benefit and are often resource-intensive [18], [19]. Robotic exoskeletons, which can safely deliver intensive therapy, have emerged as a viable tool to address some of these limitations [20].

Several exoskeletons have demonstrated potential for addressing crouch gait. For example, the Hybrid Assistive Limb has improved gait speed, cadence, and step length during in-lab training [21], while the CPWalker has shown gains in strength, gait speed, and step length [22]. Additionally, an untethered ankle exoskeleton has reduced metabolic cost of walking while worn and increased gait speed after intensive treadmill training [4], [23]. Still, long-term, low-cost training outside clinical research settings remains limited. The NIH-Agilik exoskeleton seeks to address this gap. Designed for community use, it provides torque at the knee joint to reduce crouch gait and supports both assistance and resistance paradigms for personalized training [3], [16]. The exoskeleton includes bilateral knee actuators, passive ankle mechanisms, and FSRs in the footbed for GED within a five-phase FSM controller. However, in addition to FSR durability and repeatability issues mentioned previously, force-based GED in individuals with crouch gait may be less reliable due to variations and asymmetries in gait patterns, foot placements, and weight distributions [24].

Accurate GED is paramount for exoskeletons to achieve their performance goals in clinical and community settings. For example, precise and consistent timing of extension assistance in those with crouch gait is critical to enhance knee extension and reduce metabolic cost during device use [16], [23]. When used as gait training tools in the same target population, exoskeletons must reliably apply resistive torques precisely timed to specific movements within a gait cycle (e.g., to resist ankle plantarflexion during push off [4], or knee extension during late swing [25]) to achieve functional improvements in crouch gait via strength training and motor learning. Therefore, exploration into kinematics-based GED is warranted in this population to improve exoskeleton performance and, potentially, clinical outcomes.

While shank and foot kinematics have been used for GED [8], the relatively high speed and variable acceleration of these segments during walking can lead to higher accumulated error when measured with an IMU [26]. IMU placement below the knee is also suboptimal for a single joint knee exoskeleton with all other electronics above it. To circumvent this challenge, previous studies have used thigh segment kinematics from a single thigh IMU to detect gait events in healthy and hemiplegic individuals [26], [27]. Many recent advanced GED algorithms highlighted above are not easily applied to individuals with crouch gait given the gait variability in this population. Machine-learning approaches face challenges due to highly variable gait patterns within and between participants, as well as the limited data sets in pathological gait. Consequently, GED algorithms have rarely been applied to individuals with crouch gait, and there is little evidence to indicate if thigh segment kinematics-based GED can reliably generalize to these individuals; this work aims to fill this gap. In addition to more robust control of torque delivery that can facilitate improved clinical outcomes such as increased gait speed during exoskeleton use and increased muscle strength and coordination after repeated use as a training device, IMUs also offer a promising option to quantify gait for objective assessment [28]. We hypothesized thigh segment kinematics would provide an accurate and reliable GED method for individuals with crouch gait, which could improve both rehabilitation and gait assessment capabilities outside of clinical settings.

## II. Methodology

### A. Algorithm Development

**FIGURE 1.**
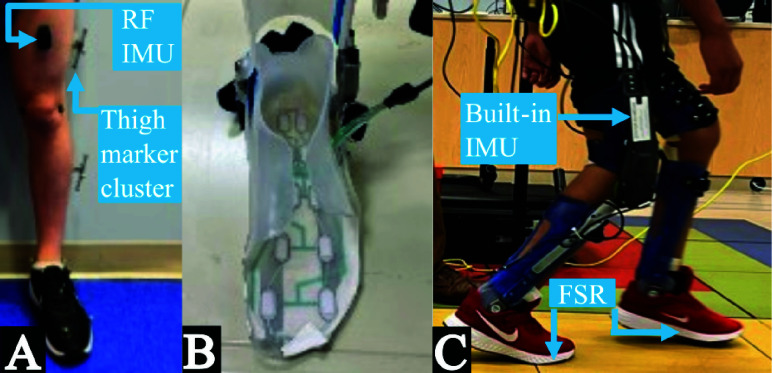
Experimental setups with an (A) IMU on the *rectus femoris* (RF) and motion capture marker configuration, (B) FSR integrated with exoskeleton knee-ankle-foot orthosis, and (C) Exoskeleton with built-in IMU and FSR.

To assess the ability of thigh segment kinematics to accurately detect gait events in individuals with crouch gait, an algorithm was created to programmatically predict IC and TC. The Thigh Segment Kinematics Algorithm (TSKA) was developed and validated through a series of steps described below. In Part I, an initial algorithm for GED from motion capture thigh segment kinematics was developed to assess the feasibility of TSKA in a population with crouch gait with the most accurate measurements possible (Fig. 1A). In Part II, the TSKA was refined for use with an IMU on the *rectus femoris* (Fig. 1A). Finally, in Part III, the TSKA algorithm was validated offline in a testbed model using an embedded IMU on the lateral thigh within the NIH-Agilik exoskeleton (Fig. 1B, 1C). Details from each Part are described in subsections II-C-E.

### B. Study Design and Participants

The TSKA algorithm was developed using previously collected data of overground walking in participants with crouch gait (Table 1) from two clinical protocols (NCT01961557, NCT05726591). During overground walking, participants completed passes covering approximately 10 meters along a straight path within the motion capture laboratory. Participants completed at least 5 passes for each condition, with breaks as requested between passes to minimize fatigue. All participants (range 6.17-16.89 years old; 7 male, 3 female; 8 cerebral palsy, 1 spina bifida, 1 muscular dystrophy) provided assent, and their respective parent(s) provided informed consent.

**TABLE 1:** Participant Demographics by Data Type

Data Source	Participant ID	Age at Enrollment (yrs)	Height (cm)	Weight (kg)	Diagnosis	GMFCS^1^	Assistive Device
Motion Capture	P1	15.37	150	46.4	Cerebral Palsy	III	Forearm Crutches
	P2	13.88	165	46.5	Cerebral Palsy	II	Walking Poles
	P3	9.84	143	41.3	Muscular Dystrophy	N/A	None
	P4	6.17	102.5	15.8	Cerebral Palsy	III	None
Rectus Femoris IMU	P5	11.15	142	39.0	Cerebral Palsy	II	None
	P6	7.39	109	18.1	Cerebral Palsy	III	Reverse Walker
	P7	16.89	161	57.4	Cerebral Palsy	II	None
Exoskeleton IMU, FSR, FSM	P8	6.41	105	16.7	Cerebral Palsy	III	Reverse Walker
	P9	11.30	145	36.2	Spina Bifida	N/A	Forearm Crutches
	P10	8.33	125	22.1	Cerebral Palsy	III	Walker with Anterior Chest Support

^1^ Gross Motor Function Classification System

### C. Part I: TSKA Development

Motion capture kinematics from overground walking at self-selected speeds in four participants were used to create the TSKA and confirm feasibility of using thigh segment kinematics for GED in participants with crouch gait. A custom 36-marker set covering the pelvis and lower extremities was used to compute segment kinematics in Visual3D (HAS Motion, Kingston, ON) [16]. IMU data were recorded at 148 Hz via Delsys Trigno system (Delsys Inc., Natick MA) and synchronized with motion capture (100 Hz) and digital video (100 Hz). Ground truth gait events (IC and TC) were manually labeled based on visual inspection of synchronized digital video. Although this introduces potential bias, force plate-based detection is unreliable in individuals using standard assistive devices (i.e., walker or crutches). A single individual conducted all labeling and was systematic across methods to minimize bias. Data were then exported to MATLAB for further analysis.

Given the proximity in timing between peak thigh angle and IC [26], an offline peak detection function in MATLAB (*findpeaks*) was used to identify local maxima of sagittal thigh angle ($\theta _{x}$). Then, a minimum threshold ($\theta _{min}$) was implemented to remove local maxima that did not correspond with an IC event, and a blanking interval prevented multiple detections of the same gait event within a specified duration ($\beta _{IC}$). These thresholding and blanking interval techniques have been shown to be effective in local peak detection algorithms [29].

Visual inspection of thigh kinematic data revealed that TC timing aligned closely with thigh angular velocity peaks for several participants, so the same peak detection and thresholding function applied to $\theta _{x}$ for IC was then applied to $\dot {\theta }_{x}$ for TC detection. Although prominent $\dot {\theta }_{x}$ peaks did align reasonably with TC events, further inspection revealed $\dot {\theta }_{x}$ often had additional peaks that did not align with TC events; these undesired peaks necessitated an additional algorithmic component to differentiate between noise and $\dot {\theta }_{x}$ peaks which aligned with ground truth TC events. Due to the multi-planar nature of walking in children with crouch gait [19], we hypothesized a weighted thigh angular velocity that combined data from the sagittal and frontal planes,

\begin{equation*} \dot {\theta }_{w} = \mathit {W}_{x} \dot {\theta }_{x} + \mathit {W}_{y} \dot {\theta }_{y}, \tag {1}\end{equation*}

could provide a more robust signal. To optimize constant weights $W_{x}$ and $W_{y}$, a grid search was performed across different weighting combinations in each plane (0 to 1, step size 0.2). For each TC event, absolute timing difference ($|\Delta _{TD}|$) between the predicted and ground truth TC was computed as

\begin{equation*} |\Delta _{TD}| = |\mathit {TC}_{Predicted}-\mathit {TC}_{GroundTruth}|. \tag {2}\end{equation*}

**FIGURE 2.**
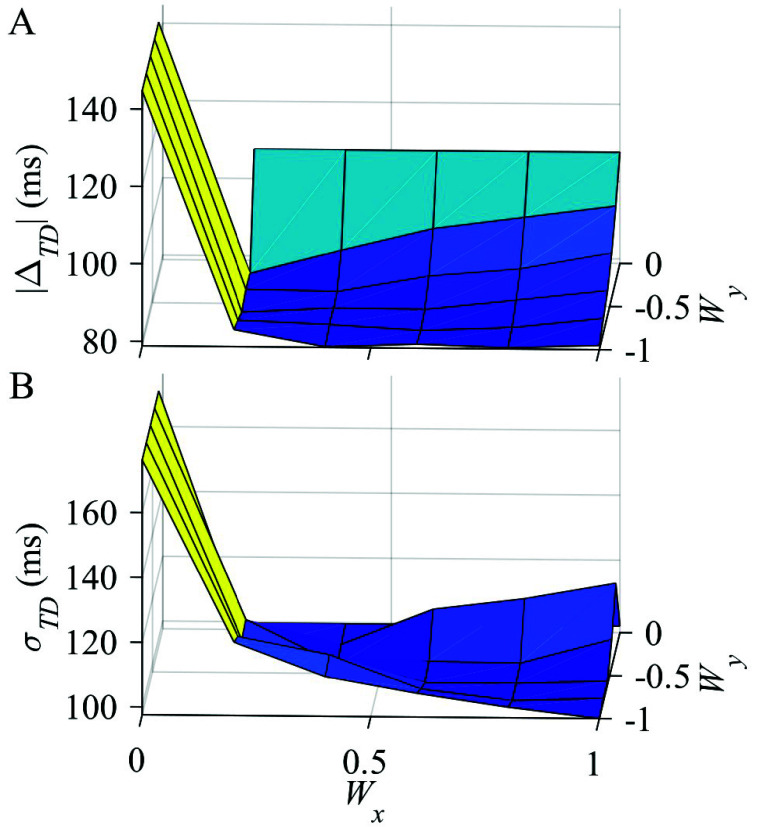
Terminal contact grid search results for (A) absolute timing difference $|\Delta _{TD}|$ and (B) standard deviation of timing difference $\sigma _{TD}$ across weighting pair combinations ($W_{x}$, $W_{y}$) for sagittal (x) and frontal (y) plane thigh angular velocity.

The optimal weighting pair was selected based on the minimization of the average $|\Delta _{TD}|$ and standard deviation of timing difference $\sigma _{TD}$ across all TC events. The weighting pair of ($W_{x}$ = 1, $W_{y}$ $= -1$) resulted in nearly the lowest average $|\Delta _{TD}|$ and lowest $\sigma _{TD}$ among weighting pairs (Fig. 2). This choice sacrificed minimal overall mean prediction accuracy but substantially decreased variability, thereby enhancing the feasibility of real-time implementation. These constants were retained in TSKA-based TC detection for the remainder of the study.

**FIGURE 3.**
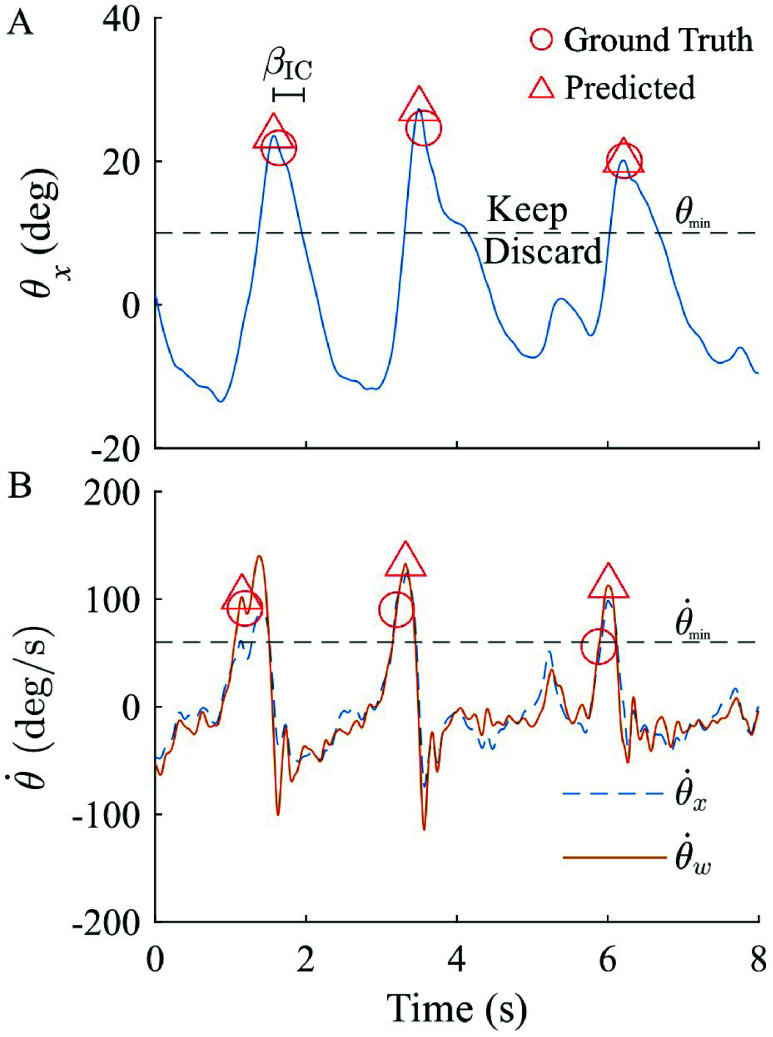
Exemplar trial demonstrating (A) IC detection and (B) TC detection. Minimum thresholds ($\theta _{min}$ and $\dot {\theta }_{min}$) in addition to a blanking interval ($\beta _{IC}$) were used to remove undesired predicted peaks. For TC detection, compared to sagittal thigh angular velocity ($\dot {\theta }_{x}$), the weighted angular velocity ($\dot {\theta }_{w}$) increases the magnitude of the desired peak at 1.15s and reduces the magnitude of the false peak at 5.22s.

This initial TSKA was implemented in participants P1-P4 and resulting IC and TC predictions were compared to ground truth, with an exemplar trial demonstrating performance shown in Fig. 3. The magnitude thresholding ensured only accurate peaks were detected (Fig. 3A), and weighting thigh angular velocity amplified peaks near ground truth TC events while reducing the magnitude of undesired peaks (Fig. 3B).

### D. Part II: Applying TSKA to Rectus Femoris IMU Data

**FIGURE 4.**
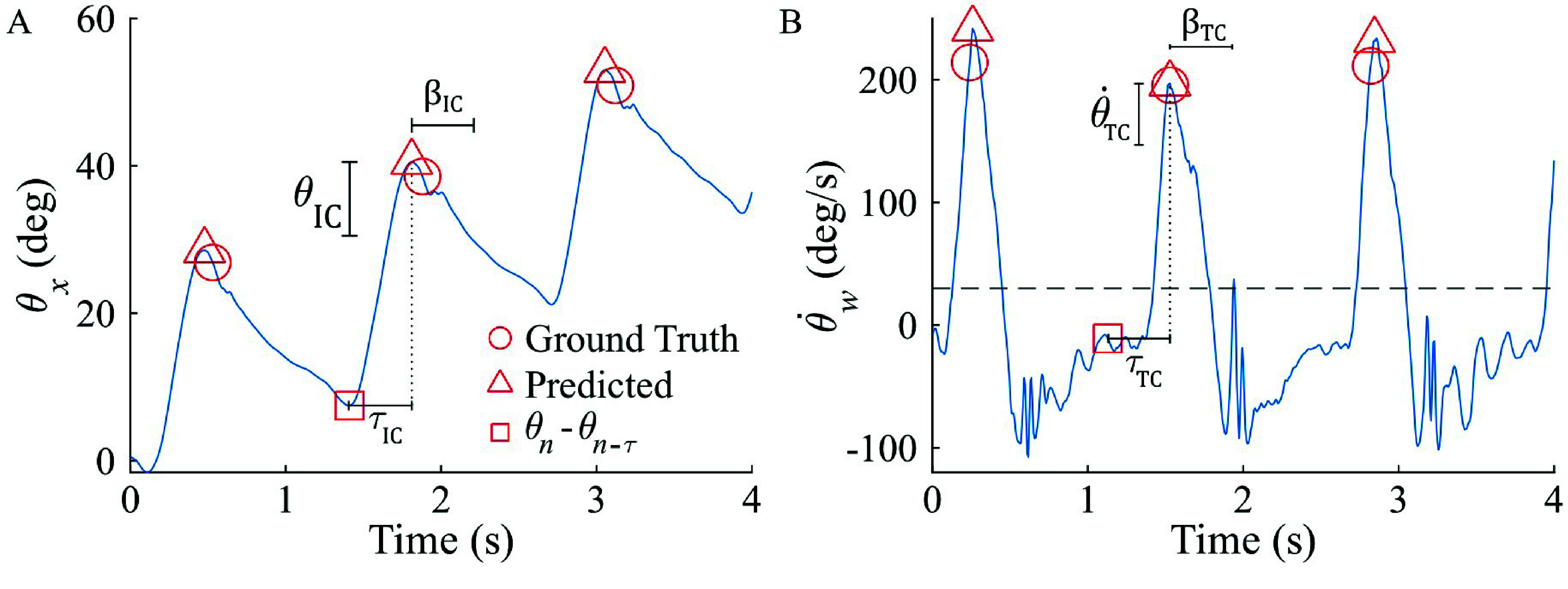
Adjusted TSKA with adaptive thresholding for (A) initial contact and (B) terminal contact detection using an IMU on the *rectus femoris*. Thresholds shown for initial contact include peak prominence ($\theta _{IC}$), timing interval ($\tau _{IC}$), and blanking interval ($\beta _{IC}$). Thresholds shown for terminal contact include peak prominence ($\dot {\theta }_{TC}$), timing interval ($\tau _{TC}$), blanking interval ($\beta _{TC}$), and peak magnitude ($\dot {\theta }_{min}$). $\beta $ and $\tau $ values ranged from 400-600 milliseconds across participants.

Several modifications to the TSKA were required for application to IMU data. Sagittal thigh angle is computed via integration of measured thigh angular velocity, yielding drift in peak magnitude over time and thus false peaks detected via only a constant minimum threshold. Additionally, IMUs contain more noise than motion capture, creating additional false peaks. An adaptive thresholding technique was developed to address these challenges (Fig. 4). For a given detected peak $\theta _{n}$ at time $t_{n}$, its magnitude relative to the value of $\theta _{x}$ at a constant time before it ($\tau _{IC}$), defined as $\theta _{n}$ - $\theta _{n-\tau _{IC}}$, was computed. The formula

\begin{align*} \begin{cases} \displaystyle \theta _{n} - \theta _{n-\tau _{IC}} > \theta _{IC}, & \text {retain} \\[3pt] \displaystyle else, & \text {discard} \end{cases} \tag {3}\end{align*}

was used to determine retention for GED. Participant-specific thresholds for both $\theta _{min}$ (IC) and $\dot {\theta }_{min}$ (TC) were tuned by visual inspection of IMU data from the first walking pass, which ranged from 2-6 strides across participants, and set between the highest non-event peak and the lowest event-related peak. Data from an IMU on the *rectus femoris* were collected from 3 participants with crouch gait to evaluate TSKA performance.

### E. Part III: Applying Finalized TSKA to Exoskeleton IMU Data

Finally, to assess feasibility in the intended clinical application, the TSKA was evaluated in three participants who walked overground with the NIH-Agilik, a previously validated lower-extremity exoskeleton that has reduced crouch gait during overground walking [25]. The device consists of a custom bilateral brace with a single actuator at each knee joint that provides bidirectional torque in the sagittal plane, a passive ankle with adjustable stiffness, a knee angle encoder, FSRs under each footbed, and an internal IMU near the thigh. Further device details can be found in [25].

The TSKA was applied to previously collected data recorded by the NIH-Agilik exoskeleton. Data were collected under an ongoing clinical protocol evaluating the exoskeleton as a gait training intervention in children with crouch gait. Participants walked in-lab using the exoskeleton with one of three torque profiles: a Zero torque profile in which the exoskeleton was set to provide a net zero torque at the knee; an Assist profile in which the exoskeleton provided torque to assist knee extension; and an Interleaved profile in which the exoskeleton provided knee extension assistance during mid-stance phase and resisted knee extension (with a flexion torque) during late swing phase [25]. Under the protocol, the torque profiles were customized to each participant, and not all participants walked with all torque profiles. Digital video simultaneously recorded through Vicon Nexus was used to label ground truth gait events. To synchronize exoskeleton and video data, the knee angle data from the exoskeleton’s encoder and motion capture were aligned using a template matching procedure that shifted the signals by averaging the timing difference between peaks [30] and was confirmed via visual inspection. For participant P10, motion capture knee angle data were unavailable due to a visual obstruction of markers, so sagittal thigh angle was used. TSKA thresholds were then tuned for each participant independently, and the algorithm was applied to the exoskeleton IMU data.

### F. Methods for Evaluation

Several metrics were computed to assess TSKA accuracy and reliability during algorithm development. ($|\Delta _{TD}|$) between a ground truth gait event and its nearest TSKA prediction and $\sigma _{TD}$ were calculated to assess accuracy and distribution of predictions, respectively. Additionally, %false positive (FP) and %false negative (FN) error rates were calculated to assess reliability. FP rates were calculated by dividing the number of extra predictions (cases with >1 prediction per ground truth event) by the total number of gait events analyzed. FN rates were computed by dividing the number of missed gait events by the total number of gait events. For both motion capture and *rectus femoris* IMU data, two generalized linear mixed effects models

\begin{equation*} \Delta _{TD} \sim 1 + (1\mid \text {Participant}) \tag {4}\end{equation*}

were used to compare $\Delta _{TD}$ to 0 in IC and TC gait events. Confidence intervals were used to assess if timing difference between predicted and ground truth gait events significantly differed and thus gauge viability of the TSKA.

To compare GED accuracy between the TSKA and current GED techniques used for exoskeleton control, gait event predictions were calculated using both the exoskeleton FSR and FSM [31] from the same data in Part III. To generate FSR-based predictions, the exoskeleton’s real-time voltage thresholds for IC and TC were applied to the raw FSR data. Details on the FSR threshold tuning are in [3]. For FSM-based predictions, IC and TC were predicted when the FSM transitioned from swing to stance and stance to swing phases, respectively. $|\Delta _{TD}|$, $\sigma _{TD}$, and error rates were calculated for FSR- and FSM-based predictions. The real-time FSM was designed to transition into waiting or standing modes if the user stopped walking. In several instances, inaccurate FSR voltages caused a false transition into waiting or standing modes when actual gait events occurred. To fairly compare the TSKA (detects only IC and TC) and the FSM (designed with higher classification dimensionality), such gait events were excluded. For statistical analysis, a generalized linear mixed-effects model

\begin{align*} |\Delta _{TD}| & \sim 1 + \text {Technique} + \text {GaitEvent} \\ & \quad + \text {Technique}*\text {GaitEvent} \\ & \quad + (1\mid \text {Participant}) + (1\mid \text {Participant:Technique}) \tag {5}\end{align*}

was used to assess fixed effects of Technique (TSKA, FSR, FSM) and GaitEvent (IC, TC), random effect of Participant (intercept), and interactions. If significant effects were observed ($\alpha < 0.05$), post-hoc analysis (F-test) was conducted.

## III. Results

### A. Part I: Motion Capture-Based TSKA Results

Participants with crouch walked at gait speeds between 0.22-1.35 m/s and cadences of 46-133 steps/min (Table 2). Motion capture-based TSKA predictions for IC events (n=770) differed from ground truth by $61\pm 30$ ms $(\text {mean} \pm \text {standard deviation})$, while TC predictions (n=676) differed by $80\pm 76$ ms. IC event predictions significantly led ground truth (p<0.001, 95% CI = [−86 ms, −49 ms]), whereas predicted TC events significantly lagged ground truth (p=3.6e-2, 95% CI = [3 ms, 86 ms]). Mean IC predictions led ground truth for all four participants (range −85 to −38 ms), while mean TC predictions lagged ground truth for three (P1, P2, P4) of four participants (range −16 to 100 ms).

**TABLE 2:** Temporospatial Metrics by Participant

Data Source		Gait Speed (m/s)	Cadence (steps/min)
Motion Capture	P1	0.22	49
	P2	0.93	94
	P3	0.84	104
	P4	0.73	133
Rectus Femoris IMU	P5	0.76	83
	P6	0.50	105
	P7	0.34	87
Exoskeleton IMU	P8	0.25	59
	P9	0.64	80
	P10	1.35	46

### B. Part II: Rectus Femoris IMU-Based TSKA Results

Timing error for TSKA-predicted IC (n=400) and TC (n=354) events from *rectus femoris* IMU data were $38\pm 55$ ms and $84\pm 74$ ms, respectively, which were similar or less than motion capture-based predictions. Consistent with Part I, IC predictions occurred before ground truth (p=8.6e-12, 95% CI = [−29, z16 ms]). However, IMU-based TSKA predicted TC events were not significantly different from ground truth (p=0.13, 95% CI = [−13, 104 ms]). Mean IC predictions led ground truth for all three participants (range −30 to −12 ms), while mean TC predictions lagged ground truth for two (P5 and P6) of three participants (range −26 to 94 ms). Several exemplar strides from *rectus femoris* IMU data are shown in Fig. 4.

### C. Part III: Exoskeleton IMU-Based TSKA Results

**FIGURE 5.**
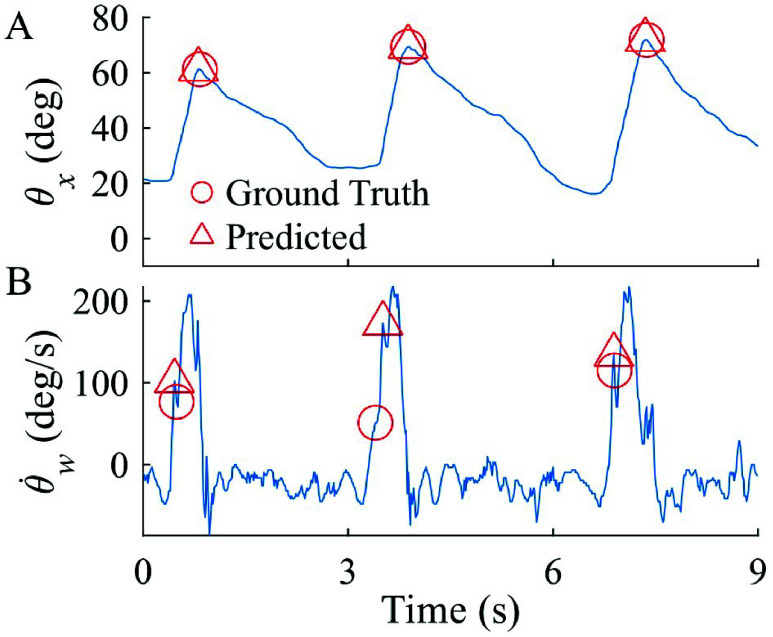
Exemplar trial showing sagittal thigh angle and weighted angular velocity from exoskeleton IMU for (A) initial contact and (B) terminal contact detection.

**FIGURE 6.**
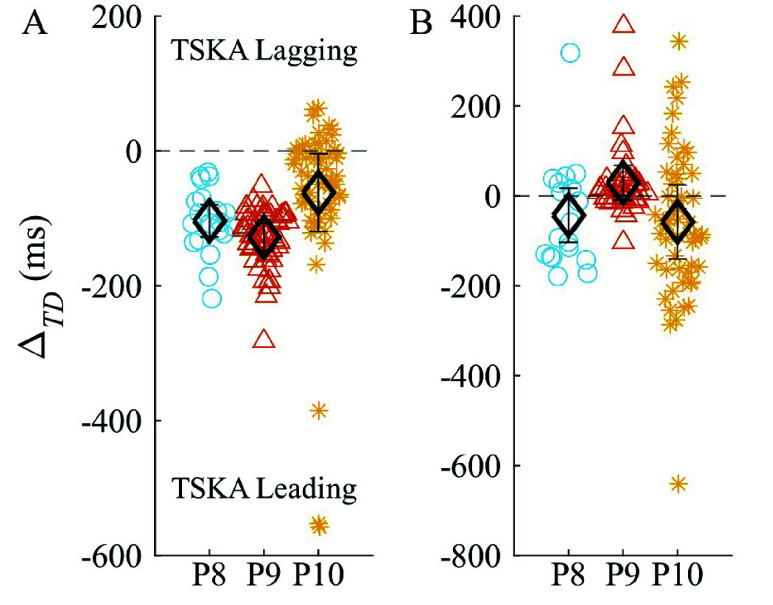
Timing differences by stride for each participant during overground walking with exoskeleton for (a) initial contact and (b) terminal contact TSKA detection compared to digital video ground truth. Diamonds and error bars indicate mean $\pm ~0.5$ std and horizontal dashed lines indicate ground truth.

TSKA performance in overground walking with the NIH-Agilik exoskeleton was consistent with *rectus femoris* IMU performance (Fig. 5), with mean $|\Delta _{TD}|$ of $100\pm 75$ ms for IC and $94\pm 125$ ms for TC (n=135 and 117, respectively) across the three participants (Fig. 6). This included 21 strides (P10) with the Zero torque profile, 37 strides (22 from P9, 15 from P10) with the Assist torque profile, and 55 strides (17 from P8, 21 from P9, and 17 from P10) with the Interleaved torque profile. The differing torque profiles applied during these trials potentially contributed to the higher mean timing differences compared to motion capture and *rectus femoris* IMU detection.

**FIGURE 7.**
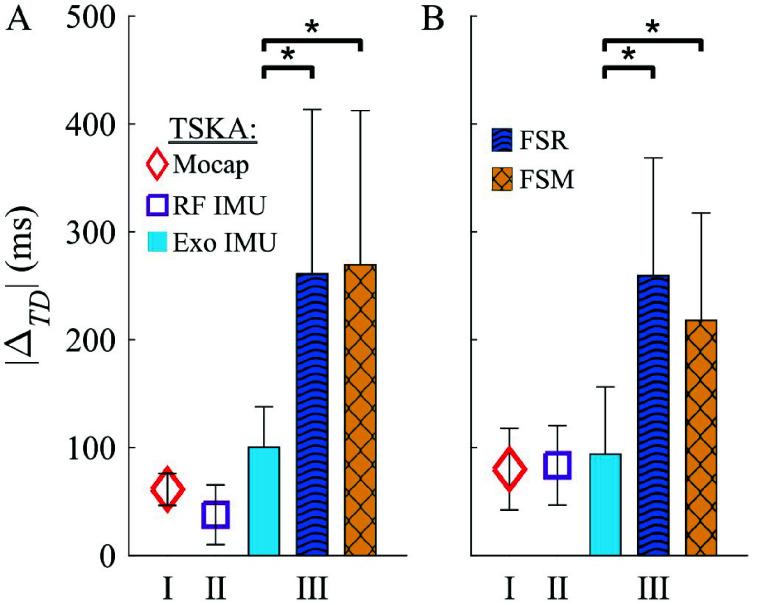
Average absolute timing differences ($\pm ~0.5$ SD) across participants for (a) initial and (b) terminal contact detection compared to ground truth video labeling. Statistical analysis compared Exoskeleton IMU, FSR, and FSM performance within the same dataset, while motion capture and *rectus femoris* IMU data had distinct datasets. FSM gait events were predicted in real-time, while FSR and TSKA predictions were generated offline (*$p< 0.001$). Motion capture and RF IMU data were analyzed in Parts I and II, respectively, while Exo IMU, FSR, and FSM data were analyzed in Part III.

For FSR-based detection, $|\Delta _{TD}|$ for IC and TC were $261\pm 305$ ms (n=128) and $259\pm 218$ ms (n=109), respectively. Finally, IC and TC absolute timing differences for FSM-based GED were $270\pm 286$ ms (n=121) and $218\pm 199$ ms (n=92), respectively. A comparison of IC and TC detection across all techniques is shown in Fig. 7.

Gait event (IC vs TC) did not have a significant effect on $|\Delta _{TD}|$ (p=0.54), but there was a significant main effect for Technique (p=3.0e-7). The TSKA significantly outperformed (lower $|\Delta _{TD}|$) FSR (p=2.0e-19) and FSM (p=8.6e-19), while FSR and FSM did not significantly differ (p=0.76). Interactions between FSR*GaitEvent and FSM*GaitEvent were not significant (p=0.70 and p=0.36, respectively).

### D. Error Rates

In addition to improved timing accuracy, TSKA FP and FN rates (Table 3) were less than 4% across all data sources (motion capture, *rectus femoris* IMU, and exoskeleton IMU). FNs were excluded from timing accuracy analysis because timing difference cannot be computed in these cases, causing FSM and FSR data to have fewer analyzed gait events than the exoskeleton IMU. Four of five detection methods (all TSKA methods and FSR) had higher FP rates for TC than IC. Two TSKA methods (TSKA - Mocap and TSKA - RFIMU) had higher FN rates for IC than TC, whereas FSR and FSM each had higher FN rates for TC than IC. Notably, FSR-based detection had the highest FP rates, while FSM-based detection had the highest FN rates. While the FSM’s additional rules dictating gait phase transitions decreased FP rates compared to simple voltage thresholding used for FSR-based GED, these rules likely contributed to the higher FN rates seen in FSM-based detection. Of 253 gait events analyzed, 37 were excluded from error rate analysis since the FSM was in waiting or standing mode.

**TABLE 3:** Error Rates Across Detection Methods

Technique	Total Events		False Positive (%)		False Negative (%)	
	IC	TC	IC	TC	IC	TC
TSKA - Mocap	770	676	1.30	3.25	0.26	0.00
TSKA - RFIMU	400	354	0.50	1.13	0.25	0.00
TSKA - ExoIMU	135	117	0.00	1.51	0.00	0.00
FSR	128	109	36.53	50.46	1.50	2.75
FSM	121	92	12.07	9.97	3.89	6.63

## IV. Discussion

To our knowledge, this is the first study to analyze gait event detection accuracy using thigh segment kinematics in a population with crouch gait. This work successfully developed and validated a novel algorithm for GED from thigh segment kinematics in this population. Previous studies have reported timing errors from sensor-based GED methods in healthy volunteers [9], [14], while others have assessed GED accuracy in a clinical population using motion capture-based kinematics of the heel and toe [8], [32]. Previous work measured GED accuracy from thigh segment kinematics in individuals with hemiplegia and healthy controls [26], and similar to [8], found kinematics-based IC predictions generally led ground truth, while TC timing error varied. Our current study found higher timing errors than those reported in previous studies of both healthy and pathological gait; however, these studies often compared predictions to force plate estimations of gait events, whereas our ground truth events were determined from digital video inspection since most participants used assistive devices, making ground reaction forces (GRFs) an unreliable GED method. Timing of events labeled from digital video inspection will inherently differ from GRF-based labeling due to the timing difference between ground contact and weight loading/unloading. This may have led to worse accuracy compared to previous work but is a more conservative approach for measuring error. This work builds upon prior studies by validating thigh segment kinematics-based GED in a population with crouch gait. The viability of the TSKA for detecting IC and TC events establishes the feasibility of using thigh segment kinematics to control wearable robotics and rehabilitation devices in this population, including the subgroup who use assistive gait aids such as walkers or crutches.

While many rehabilitation devices, including the NIH-Agilik exoskeleton, use force-based sensors underfoot to detect IC and TC, our results suggest the TSKA can detect these gait events with significantly higher accuracy in children with crouch gait. Specifically, TSKA-based predictions differed from ground truth IC and TC events by an average of <100ms, with only 12 outliers among all 1446 gait events analyzed (Fig. 6), while FSR- and FSM-based predictions differed by >200ms. This difference between TSKA and FSR/FSM techniques exceeds the ground truth resolution (10 ms per frame) by 10-fold, suggesting a reliably improved detection performance. Importantly, TSKA error rates were less than 4% across all three data sources, whereas FSR- and FSM-based detection exhibited higher error rates. FSR-based detection was the most sensitive, with IC and TC FP rates above 35%. Conversely, the FSM had fewer FPs than FSR but had the largest FN rates, with both IC and TC above 3.8% compared to <2.8% in all other techniques. The improved accuracy and lower FP and FN rates of TSKA-based GED compared to the current FSM of the NIH-Agilik exoskeleton provides impetus for future exploration into utilizing thigh segment kinematics for exoskeleton control. Despite the simplicity of the TSKA, our results suggest that the algorithm may be more effective than current portable FSR-based GED methods in children with crouch gait. While future study with more advanced models such as machine-learning based methods is warranted, the TSKA algorithm is easily implementable with little computational burden. Furthermore, personalized tuning was achieved using visual observation of data from at most 6 strides (Fig. 3), compared to machine learning GEDs which require large training datasets across many users [15], [33], [34]. This fast, intuitive tuning could be completed directly by clinicians to enable straightforward translation to clinical and real-world settings, and is particularly important during use of an exoskeleton which alters a user’s gait pattern. Given that accurate GED is essential to maximize user benefit [7], employing the TSKA could potentially increase clinical outcomes of the NIH-Agilik exoskeleton by improving the consistency and timing of torque delivery during use. When used as an assistive device, this enhanced accuracy could further increase gains in knee extension and gait speed while walking with the exoskeleton [16]. During gait training in clinical and community settings, the improved GED would improve the timing of resistive torque delivery, thereby potentially improving therapeutic outcomes resulting from increased muscle activation during periods of applied resistance [25].

Two major factors contributed to the observed higher error rates in FSR- and FSM-based GED compared to those published in a previous analysis of the NIH-Agilik FSM [3]. First, all participants in this analysis (P8-P10) used weight bearing assistive devices (walker or crutches), resulting in some fraction of their weight being supported by the arms during walking, which likely caused inconsistent or delayed loading onto the FSRs. Consequently, errors in FSR- and FSM-based detection were likely exacerbated compared to the previous study in which minimal body weight support was applied through the arms during walking. Second, while the FSM subdivides the gait cycle into five phases, only two gait events (IC and TC) were examined in this study. The FSM’s control laws for these two events require FSR input; therefore, inaccuracies in FSR-based detection for this population likely skewed the FSM results to appear less accurate than previously reported, which aggregated accuracy across all five phases (i.e., three that do not require FSR data) [3]. Substituting the current FSR-based GED with the TSKA could enhance robustness of the overall FSM including and especially during use of mobility aids like walkers or crutches, in turn maximizing potential benefit of rehabilitative exoskeletons.

Previous work has found FSR-based GED to be highly accurate in healthy individuals (mean timing error 1.5 ms compared to force plate detection [14]). Our results demonstrate substantial delay in participants with crouch gait using mobility aids like walkers and crutches. It should be noted these delays represent a smaller percentage of the gait cycle due to slower gait speed (Table 2). Several factors likely influenced these results. First, delays in foot loading/unloading from atypical walking patterns and mobility aids would delay gait event predictions, accentuating error compared to visual identification of foot contact. Previous studies have shown individuals with crouch gait exhibit uneven foot placement and weight distribution during gait, making force-based measurements less accurate for this population [24]. As mentioned previously, these delays are likely accentuated when using assistive devices since a portion of body weight is loaded onto the device instead of the force sensors, as was the case here. This issue further supports use of the TSKA, which is agnostic of weight loading/unloading. Additionally, errors likely stemmed from the FSR hardware itself; these sensors can often exhibit inconsistent voltage and cell degradation in repeated use, which contribute to GED inaccuracy, requiring frequent recalibration and occasionally replacement [13]. Because many rehabilitation devices use similar FSR-based GED, FSR fragility could limit long-term therapeutic potential. Alternatively, thigh segment kinematics-based detection may provide similar or even improved GED accuracy without requiring FSR hardware. Moreover, this GED method can minimize exoskeleton device footprint with a more proximal IMU rather than on or near the foot. Therefore, the TSKA may provide a simpler, more robust controller of exoskeletons and rehabilitative devices for populations with crouch gait, improving their viability for use outside clinical environments.

There are multiple limitations to the work presented. Although real-time execution was simulated, the TSKA was implemented offline. Future testing of real-time gait event detection with TSKA is necessary to integrate the technique into a closed-loop controller. The NIH-Agilik exoskeleton already contains a thigh-mounted IMU, enabling the TSKA to be implemented in real-time with no change in hardware. The *findpeaks* algorithm uses a sliding window to examine if a peak has occurred by detecting a change in the sign of the time series slope. A similar algorithm has already been implemented in the NIH-Agilik FSM to detect a change in knee angle direction [25]. Thus, the same functionality could be applied in real-time with the IMU to implement TSKA with a minimal amount of latency equal only to the sliding window size. In our offline testing window size was two samples which would translate to a latency of 20 msec if used online. One possible failure mode is that noise in the IMU would require filtering and/or a longer sliding window to distinguish peaks more reliably; this should be carefully examined in future experiments. Because at most six strides were required for tuning, in a clinical workflow, a visual display of the thigh angle and angular velocity data could be built into the software application used for exoskeleton setup and personalization (which already includes the capability to display real-time sensor data for exoskeleton FSM tuning [13], [25]) to enable inspection of peak detection accuracy by the clinician. Second, data from only 3-4 participants were used to evaluate TSKA in each data source. Whereas these results support feasibility of the algorithm, given the variability in gait patterns in people with crouch gait, further study with more participants is warranted. Additionally, future studies encompassing a wider range of GMFCS levels (I-IV) and diagnoses could elucidate the algorithm’s effectiveness across the broad target population thereby informing clinical deployment outside the research setting.

The TSKA has potential applications beyond enhancing rehabilitative device control. Implementation only requires a single IMU on each thigh with straightforward logic to accurately predict initial and terminal contact. Ground contact events enable temporal gait analysis in multiple settings (e.g. stride time, stride asymmetry, swing vs stance phase duration, or cadence), valuable metrics to assess gait [35]. Our findings support validity of obtaining these measures in children with crouch gait from thigh-mounted IMUs, potentially lowering barriers to study crouch gait and its progression outside clinical settings with relatively low cost. Overall, the ability of the TSKA to accurately predict gait events in people with crouch gait has the potential to be used for a variety of robotic control systems and gait assessment tools.
